# A PaDd-informed, Assay-calibrated Diagnostic Pathway for Suspected Pulmonary Embolism in Older Adults: From Signal to Strategy

**DOI:** 10.5041/RMMJ.10568

**Published:** 2026-01-28

**Authors:** Mulavagili Vijayasimha, Sivaji Ganesh Adusumalli

**Affiliations:** 1 Department of Medical Laboratory Technology (Biochemistry), University Institute of Allied Health Science, Chandigarh University, Mohali, Punjab, India; 2 Department of Biochemistry, Acharya Nagarjuna University, Guntur, Andhra Pradesh, India

**Keywords:** Assay calibration, clinical decision rules, D-dimer, diagnostic pathways, geriatric medicine, PaDd algorithm, pulmonary embolism, risk stratification

## To the Editor

We commend Cohen et al. for introducing a pragmatic composite—Padua score × D-dimer (PaDd)—designed to refine pulmonary embolism (PE) exclusion in adults aged ≥65 years, a population often characterized by multimorbidity, physiological heterogeneity, and atypical presentations. Their single-center retrospective cohort (2021–2023) provides a compelling, hypothesis-generating signal: combining a validated venous thromboembolism risk score with D-dimer may enhance specificity without compromising safety.^1^

### Context of Existing Evidence

Two decades of research confirm that adaptive D-dimer strategies consistently and safely reduce imaging utilization. Age-adjusted D-dimer (AADD) and clinical-probability-adapted models are now well established.^2^ The two algorithms—Pulmonary Embolism Graduated D-dimer (PEGeD) and YEARS (based on three clinical criteria: signs of deep vein thrombosis, hemoptysis, and pulmonary embolism as the most likely diagnosis)—integrate pre-test probability with dynamic D-dimer thresholds, maintaining low miss-rates while significantly reducing CT pulmonary angiography (CTPA) exposure across emergency and primary-care settings.^3^,^4^

Against this backdrop, PaDd is appealing because it encodes comorbidity-driven thrombotic risk and leverages D-dimer’s high sensitivity. However, transforming PaDd from a promising signal into an implementable diagnostic policy requires attention to four critical domains.

1. Comparator-anchored validation: PaDd’s performance must be benchmarked directly against AADD, YEARS, and PEGeD using identical reference standards and 3-month venous thromboembolism outcomes. External validation of PEGeD has already revealed contexts in which 1000-ng/mL thresholds may be insufficient, particularly above age-adjusted limits—highlighting gaps a PaDd-augmented model should proactively address.^3^
2. Assay-specific calibration: D-dimer is not a single test, and assay heterogeneity meaningfully affects cutoff performance. Data from the ADJUST-PE study show wide inter-assay variability when AADD is applied.^5^ A future PaDd rule must therefore be assay-calibrated, with outcomes stratified by reagent platform to ensure reproducibility and clinical safety.
3. System-level and ethical considerations: In emergency-care systems worldwide, over-testing, under-testing, and mis-testing of suspected PE persist.^6^ Reducing low-value CTPA is a clinical, economic, and ethical imperative. A PaDd pathway should be embedded within a de-implementation framework supported by decision-curve analysis, cost-effectiveness modeling, and equity metrics, especially in resource-constrained settings.
4. Downstream management of subsegmental PE: Diagnostic parsimony should align with therapeutic parsimony. Structured surveillance without anticoagulation is safe for carefully selected patients with isolated subsegmental PE, yet remains underutilized.^7^ A PaDd-based pathway should predefine subsegmental PE management contingencies to avoid inadvertently replacing imaging overuse with treatment overuse.

### Proposed Validation Roadmap

We propose a four-part roadmap to strengthen the next stage of PaDd evaluation:

- Pre-test probability: Classification using YEARS or 4PEPS (4-Level Pulmonary Embolism Clinical Probability Score) where validated.^4^,^8^,^9^
- Assay-calibrated D-dimer: Application of assay-specific AADD or clinical-probability-adapted thresholds.^2^,^5^
- PaDd overlay for ≥65 years: Adjustment of D-dimer cutoffs upward for low comorbidity (low Padua score) and downward for high-risk profiles.^1^
- Equity and safety framework: Reporting calibration accuracy, safety margins, and failure rates stratified by age, estimated glomerular filtration rate (eGFR), cancer status, and assay type.^3^,^5^ Integration of subsegmental PE management aligned with local follow-up capacity.^7^,^8^

### Global Significance

If validated rigorously, assay-informed PaDd-augmented pathways could meaningfully reduce imaging in regions where scanners are scarce, contrast nephropathy is prevalent, or workforce capacity is limited. Evidence from primary-care YEARS already demonstrates feasibility; integrating PaDd for geriatric patients may further enhance diagnostic equity and safety.^4^

Cohen et al. have introduced a clinically relevant and geriatric-sensitive concept.^1^ To ensure that PaDd becomes not only innovative but implementable, future studies must be multicenter, assay-calibrated, transparent, and anchored to comparator trials. Such rigor will help deliver a diagnostic pathway that is safer, scalable, and ethically aligned with global standards.

## Abbreviations

AADD: age-adjusted D-dimer
CTPA: computed tomography pulmonary angiography
PaDd: Padua score × D-dimer
PE: pulmonary embolism
PEGed: Pulmonary Embolism Graduated D-dimer
YEARS: algorithm based on three clinical criteria: signs of deep vein thrombosis, hemoptysis, and pulmonary embolism as the most likely diagnosis.
